# A Comparison of Bottom-Up Models for Spatial Saliency Predictions in Autonomous Driving

**DOI:** 10.3390/s21206825

**Published:** 2021-10-14

**Authors:** Jaime Maldonado, Lino Antoni Giefer

**Affiliations:** Cognitive Neuroinformatics, University of Bremen, Enrique-Schmidt-Straße 5, 28359 Bremen, Germany; l.giefer@uni-bremen.de

**Keywords:** autonomous driving, bottom-up saliency models, perception, saliency detection, saliency maps, visual salience

## Abstract

Bottom-up saliency models identify the salient regions of an image based on features such as color, intensity and orientation. These models are typically used as predictors of human visual behavior and for computer vision tasks. In this paper, we conduct a systematic evaluation of the saliency maps computed with four selected bottom-up models on images of urban and highway traffic scenes. Saliency both over whole images and on object level is investigated and elaborated in terms of the energy and the entropy of the saliency maps. We identify significant differences with respect to the amount, size and shape-complexity of the salient areas computed by different models. Based on these findings, we analyze the likelihood that object instances fall within the salient areas of an image and investigate the agreement between the segments of traffic participants and the saliency maps of the different models. The overall and object-level analysis provides insights on the distinctive features of salient areas identified by different models, which can be used as selection criteria for prospective applications in autonomous driving such as object detection and tracking.

## 1. Introduction

Visual attention is the mechanism by which human beings can selectively process salient stimuli. The selection mechanism can be influenced by *bottom-up* and *top-down* factors [1]. Bottom-up factors refer to the features of the image (e.g., color, intensity and orientation). Top-down refers to the cognitive factors of the observer which determine whether an object or a region of the visual field is salient. In the context of driving, different regions of a street are salient depending on the task, e.g., whether the driver is looking for a parking spot or just driving forward.

**Figure 1:**
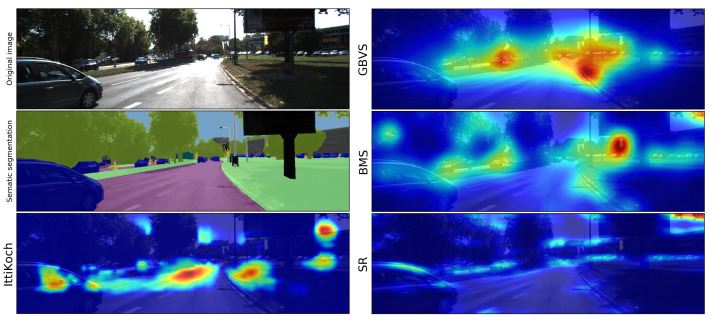
Example of an image, its semantic segmentation and the corresponding saliency maps obtained with different bottom-up models. The example illustrates how salient areas identified with different models differ on their shape, size, location and saliency level. The models are described in Section 2.

Bottom-up factors have been extensively studied in the literature and many computational models to identify salient regions have been proposed [1]. Depending on the computational mechanisms involved, as well as the features or cues used to detect saliency, different bottom-up models identify salient areas of an image differently (see Section 2). As a result, salient areas identified by different models differ in terms of their size, shape and location. Furthermore, an area identified as salient by one model can be regarded as non-salient by another model. These differences are illustrated in Figure 1, showing the saliency maps of an image generated by different bottom-up models.

Bottom-up saliency models have been used in computer vision applications including object detection and recognition, robot navigation and localization, and image processing (for a comprehensive list of applications see [1]). Such computer vision tasks can be encountered on applications of Advanced Driver Assistance Systems (ADAS) and highly autonomous driving (HAD). In these applications, an input image is initially processed with a bottom-up model to obtain a saliency map. As a result of the feature extraction conducted by the saliency model, the salient regions indicate the locations of proto-objects, which represent coherent areas that approximate whole, partial or groups of objects in the image. Subsequent processing steps focus on the salient areas. The incorporation of bottom-up models in object detection, object segmentation and object recognition applications enables a faster processing of the image compared to approaches that analyze the full scene to detect the objects by, for example, shifting analysis windows [2]. In the field of bottom-up saliency for object detection, Silva et al. [3] proposed a method to improve detection performance and execution speed. Specifically, saliency was used to prune the search space for objects. They evaluated their approach over a dataset for person detection in different types of scenes including cities and outdoor landscapes. They emphasize the fact that bottom-up saliency can be affected by uncontrolled factors in scenes, such as variations in color, size, illumination and noise of the target objects.

Differences in performance resulting from the use of different bottom-up saliency models have been investigated for object detection [4] and driver gaze prediction applications [5]. Duthon et al. [4] evaluated different bottom-up saliency models to test their applicability for object detection in autonomous vehicles. The experimental results show that bottom-up saliency on its own is not sufficient for robust detection in the road context. In this case, detection is not reliable because not all relevant objects (e.g., cars, bicycles or pedestrians) are necessarily salient. Nevertheless, they emphasize that bottom-up saliency can facilitate detection when used as a pre-processing step. They also conclude that factors such as the task and the context (e.g., type of landscape, point of view) can influence the performance and that the selection of a saliency model should consider the target application.

Deng et al. [5] evaluated the use of different state-of-the-art bottom-up saliency models for traffic saliency detection. The experimental results obtained from the comparison of the predicted saliency and human gaze data indicate that bottom-up saliency models cannot be directly applied to predict the drivers’ allocation of visual attention. They show that this limitation can be resolved by adding a top-down control to modulate the bottom-up prediction.

Typically, saliency models are evaluated using benchmark datasets for object detection [6] and human gaze allocation during free-viewing tasks [7]. Benchmark datasets aim at testing the applicability of saliency models over images with diverse characteristics: natural indoor and outdoor scenes, artificial stimuli (e.g., patterns), and a wide variety of objects and settings. On the contrary, context- and task-specific evaluations reveal how the models perform over images of particular characteristics and/or attention tasks. This is exemplified in the evaluation performed by Deng et al., where saliency models that are successful at predicting gaze allocation during free-viewing of images across different categories fail to predict where drivers look at while observing driving scenes [5].

To use a bottom-up saliency model in a particular autonomous driving application, it is important to consider the characteristics of the images registered by the frontal camera of a vehicle. Frontal camera images provide panoramic views of the road ahead in which the low-level features of the image, such as color, contrast and object size, might differ a lot depending on the landscape (e.g., urban or rural roads), traffic conditions (e.g., light or heavy traffic), time of day (e.g., day or night) and weather conditions. Previous evaluations of bottom-up saliency models in driving applications have been focused on task performance (i.e., driver gaze prediction or object detection). However, those studies provide no insights on how the distinctive features of the saliency predictions produced by different models influence the outcome.

In this paper, we conduct a systematic evaluation of the saliency maps computed with different bottom-up models on urban and highway scenes. Our goal is to compare the size and shape of the salient areas identified by different models and the extent to which traffic participants in the image fall within them. For this purpose, we compute the saliency of the images available in the KITTI semantic instance segmentation dataset [8]. The evaluation is based on energy and entropy features which represent the size and shape-complexity of the salient areas. In addition, we assess the agreement between the salient areas and the segments corresponding to traffic participants in the images. The features used to assess the saliency maps enable us to perform a comprehensive quantitative and qualitative comparison of different models. It is important to note that we consider the saliency computation as the initial step of a computer vision processing pipeline. Therefore, the evaluation features and criteria are not restricted to the outcome of a particular computer vision task, such as object segmentation or recognition, which depend on how the identified salient locations are processed. In this sense, our analysis describes and compares the salient areas identified in a prospective initial processing step with respect to the traffic participants in the picture. To the best of our knowledge, no other studies have been devoted to compare the size and shape complexity of salient areas and their relation to object instances in the image. The analysis aims to provide insights on how distinct salient areas are identified by different models, both over the whole image as well as on the object level. We encounter significant differences between the models which indicate the extent to which object instances (such as cars or pedestrians) fall within the salient areas.

The rest of the paper is organized as follows. In Section 2, we provide details about different types of bottom-up saliency models and describe the main characteristics of the ones that our study is based on. Section 3 focuses on details about the dataset we employ, and the computation of saliency maps followed by a thorough evaluation of those based on their energy, entropy and the agreement between the salient areas and the segments corresponding to traffic participants in Section 4. Based on the evaluation, we discuss how our results provide selection criteria to choose a particular saliency model for prospective autonomous driving applications in Section 5.

## 2. Bottom-Up Saliency Models

Based on their computational mechanisms, bottom-up models can be classified into four categories [9]: (1) *Rarity/Contrast-Based*, which compute center–surround contrast and/or local rarity/contrast based on image features, (2) *Spectral Analysis Models*, which are based on the frequency spectrum of the image, (3) *Learning-Based Models*, in which machine learning models are trained using low, middle and high-level features and/or eye tracking data, and (4) *Salient Object-Detection Models*, which aim to segment boundaries of salient objects by highlighting overall foreground regions.

In general, a saliency model takes an image I(x,y) with pixel coordinates *x* and *y* as input and outputs a saliency map S(x,y) representing the conspicuity or saliency at every location in the image by a scalar quantity [10]. Thus, saliency maps are typically displayed as intensity images, where the intensity of each pixel represents its probability of belonging to salient regions or objects. Although models belonging to the first three categories usually identify sparse blob-like salient regions aimed to predict gaze fixations, salient object-detection models often generate smooth connected areas [6].

In this work, four bottom-up models are evaluated for comparison covering three of the above-mentioned categories. In the rarity/contrast-based category we consider the IttiKoch model [10], which is based on feature integration theory, and the Graph-Based Visual Saliency (GBVS) [11], which is based on graph theory [2]. Both models perform low-level feature extraction and integration. In the spectral analysis category, we include the Spectral Residual (SR) model [12]. Finally, the Boolean Map Saliency (BMS) model [9] is considered for the salient object-detection category. The selected models are representative of each category and are frequently cited in the literature. Furthermore, three of them (IttiKoch, GBVS and SR) have been analyzed in previous studies in the context of traffic saliency detection [5] and two of them (GBVS and SR) in the context of object detection for autonomous vehicles [4]. To conduct a fair comparison, we exclude learning-based approaches as they depend on the quality of the training data and the learning capabilities of the machine learning model. In the following, we describe the main characteristics of the selected models.

### 2.1. IttiKoch: Rarity and Center—Surround Contrast Model

The model proposed by Itti et al. [10] is regarded as the baseline saliency model [2] and is referred to as the *Itti* or *IttiKoch* model in the literature. The model computes feature maps from an image’s intensity, color and orientation, which represent the feature at every location of the image by a scalar quantity [10]. These low-level features are known to attract human visual attention [2]. These feature maps are analyzed in different scales (i.e., in different resolutions) to account for objects and locations of different sizes.

The effect of the intensity, color and orientation on the saliency of a pixel region depends on the contrast with its surroundings. Thus, the edges of objects in the image, as well as regions that locally stand out from their surroundings, are highlighted by means of a center–surround operation [2]. The resulting saliency map represents local saliency over the entire image [10]. Itti et al. pointed out the applicability of the identified salient image locations for subsequent computer vision tasks, such as object detection [10].

### 2.2. Graph-Based Visual Saliency (GBVS): Rarity-Based Model

The GBVS model proposed by Harel et al. [11] extracts intensity, color and orientation features maps such as the IttiKoch model but omits the center–surround process for orientation maps and reduces the number of scales with which the image is processed [2]. As a result, the GBVS model groups sparse edges into integral regions that stand out from their surroundings [2]. GBVS produces high-saliency values in the center of the image plane (center bias) and is regarded to be robust with respect to differences in the sizes of salient regions [11].

Harel et al. argue that the center bias of the model is well suited for predicting human gaze allocation based on two observations: (1) everyday life head motion often results in gazing straight ahead and (2) the motif of photographs is typically located in the center [11].

### 2.3. Spectral Residual (SR): Spectral Analysis Model

The SR model proposed by Hou and Zhang [12] aims to simulate the behavior of pre-attentive visual search, in which low-level features such as orientation, edges, or intensities stand out automatically. In the SR model, the spatial frequency content of an image represents novel and redundant information, where a peak in the frequency spectrum is considered novel information. Based on this assumption, SR approximates the salient parts of an image by removing the statistical redundant components. The saliency map is computed based on the spectral residual of the image in the frequency domain, based on the hypothesis that saliency is the residual difference between the spectrum and the characteristic spectrum of natural images [2].

Since SR removes the statistical redundant components of an image, the saliency map highlights the non-trivial regions of the scene and suggests the positions of proto-objects [12]. SR is regarded by its authors as a general-purpose saliency detection system, well suited for object-detection applications since the saliency computation does not rely on features, categories, or other prior knowledge about the objects [12]. In contrast to the IttiKoch and GBVS models, the saliency computation of the SR model does not need to compute color, intensity and orientation feature maps or to analyze the image at different scales.

### 2.4. Boolean Map Saliency (BMS): Enclosure-Based Figure-Ground Segregation

The BMS model proposed by Zhang and Sclaroff [9] detects salient regions with closed outer contours based on the surroundedness (enclosure) cue for figure-ground segregation. As a result of the surroundedness cue, the model does not assign high-saliency values to high-contrast boundary areas typical of natural images (e.g., the boundary between the trees and the sky). Thus, compared to other models, BMS is less responsive to the edges and cluttered areas in the background [9]. Another effect of saliency detection based on the surroundedness cue is that the model highlights the interior regions of salient objects [9]. BMS can identify salient regions of different sizes due to the scale-invariant nature of the surroundedness cue and thus does not require the image to be processed at different scales [9].

BMS was proposed as a saliency detection model for eye fixation prediction but has been shown to be useful for salient object detection [9], and for the detection of proto-objects [6] outperforming several salient object-detection models [6]. Furthermore, it was ranked as the best non-neural network model on the MIT300 Benchmark [7].

To demonstrate the qualitative differences between the output generated by different models, we provide an example of the saliency maps computed for a video sequence of the KITTI object tracking dataset in Supplementary Materials.

## 3. Materials and Methods

We analyze the saliency maps obtained from semantically annotated images from urban and highway scenes, where we compute four maps from each image with the IttiKoch, GBVS, SR and BMS models. From each saliency map, we compute two features that describe the amount and shape-complexity of the salient areas. Subsequently, we assess the agreement between the salient areas and the segments corresponding to the traffic participants by means of an error measure. Finally, we compute the proportion of salient pixels for different saliency thresholds. This enables us to compare the size of the salient areas identified by different models both over the whole image, as well as on the object instance level. The statistical analysis of the differences between bottom-up models was performed with R [13] and the package *PMCMR* for pairwise comparisons [14].

### 3.1. Dataset and Computation of Saliency Maps

For the evaluation and comparison of the saliency maps produced by the selected models, we use the KITTI semantic instance segmentation dataset [8]. We choose this dataset because of the variety contained in the images with respect to classes of traffic participants (pedestrians and different types of vehicles), their position and size, traffic situations (from a single to several vehicles) and environments (urban, rural and highway scenarios). Specifically, we compute the saliency of the set of 200 training images, and we use the pixel-level semantic instance segmentation annotations to assess the agreement between the salient areas and segmented traffic participants in the image. We use the term *traffic participants* to refer to the following object categories annotated in the dataset: car, truck, person, bicycle, rider, bus, train, motorcycle, caravan, and trailer.

Saliency maps of the IttiKoch, GBVS and BMS models are computed using Pysaliency [15], a Python package for saliency modeling. The saliency maps of the SR model are computed using the OpenCV implementation (library version 4.0.1) [16]. We compute the SR with a resolution parameter of 64 pixels, which provides a good estimation of the scale of normal visual conditions [12]. An example of the saliency maps computed with the selected models is shown in Figure 1. The saliency maps produced by the IttiKoch and the BMS models are normalized such that 0≤S(x,y)≤1.

### 3.2. Description of the Amount and Shape-Complexity of Saliency Maps Based on Energy and Entropy Features

The saliency maps produced by different models are compared in terms of their energy and entropy, as defined by Perrin et al. [17], which represent the amount and shape-complexity of the salient areas in a saliency map. Based on the observation that salient areas identified with different models differ in their shape, size and saliency level, we use the energy and entropy features in this paper to provide an objective comparison between the saliency maps obtained with different bottom-up models. Our use of the energy and entropy features differs from the approach in [17], where these measures are used to assess human saliency maps (i.e., saliency maps computed from human gaze data) for the evaluation of content-wise biases of images acquired by unmanned aerial vehicles.

The energy E(x,y) of a saliency map S(x,y), defined in (1), is the sum of the vertical and horizontal gradient absolute magnitudes [17]. A Sobel filter [18] of kernel size 5 is used as derivative operator, following the implementation in [17].

(1) $$E\left(x,y\right)=\left|\frac{\partial S(x,y)}{\partial x}\right|+\left|\frac{\partial S(x,y)}{\partial y}\right|$$

**Figure 2:**
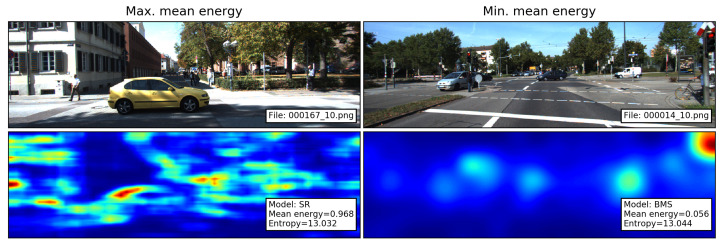
Images and their corresponding saliency maps with the maximum and minimum mean energy. SR produces several salient regions with complex shapes. In contrast, BMS produces blob-like salient areas.

As energy feature, we take the mean of E(x,y) over all pixels of the image [17]. Following the interpretation of Perrin et al., high mean energy indicates that the saliency map contains several salient regions or shape-wise complex areas of interest, whereas low energy indicates more simple-shaped salient regions [17]. To illustrate this interpretation, the saliency maps with the maximum and minimum mean energy are shown in Figure 2.

The Shannon entropy [19] *H* of a saliency map is defined in (2), where p(x,y) is the probability of a pixel to be salient.

(2) $$H=\sum_{x,y}p\left(x,y\right)\log\left(p\left(x,y\right)\right)$$

**Figure 3:**
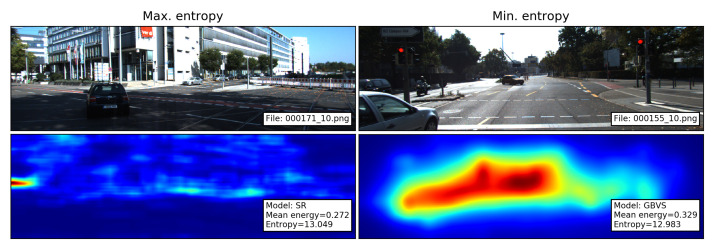
Images and the corresponding saliency maps with the maximum and minimum entropy. SR produces complex areas of saliency (cf. SR saliency map in Figure 2). In contrast, the higher saliency values produced by the GBVS model appear to be concentrated in a single zone.

A saliency map S(x,y) is converted into a probability distribution using a soft-max function:

(3) $$p\left(x,y\right)=\frac{\exp S(x,y)}{\sum_{x,y}\exp S\left(x,y\right)}$$

such that 0≤p(x,y)≤1 and $\sum_{x,y}p\left(x,y\right)=1$. Entropy has been used as feature for the evaluation of images and saliency maps [20,21], where larger entropy values indicate the presence of a complicated texture or structure [17,21]. Following the interpretation of Perrin et al. [17], a high value of entropy indicates that the saliency map contains a lot of information (i.e., “it is likely that saliency is complex”) and a low entropy value indicates a single zone of salience. This interpretation is illustrated with the saliency maps with the maximum and minimum entropy in Figure 3.

### 3.3. Evaluation of the Agreement between the Salient Areas and the Traffic Participants

Different measures have been proposed to measure the agreement between saliency predictions and annotated ground-truth. Two approaches are distinguished in the literature [6]: (1) metrics based on the overlap between salient areas identified by a model and the ground-truth (i.e., a marked region in the image), and (2) measures of the accuracy of the saliency maps with respect to the boundaries of the ground-truth regions.

To measure the agreement between a saliency map and ground-truth regions the Mean Absolute Error (MAE) is recommended over other overlap-based measures as it considers both true positives (i.e., the pixels correctly marked as salient) and the true negatives (i.e., the pixels correctly marked as non-salient) in a single measure [6,22]. (For a review of other standard and universally agreed measures in the context of object detection see [6,22], and in the context of human gaze prediction see [23].) (For a review of other standard and universally agreed measures in the context of object detection see [6,22], and in the context of human gaze prediction see [23].) (For a review of other standard and universally agreed measures in the context of object detection see [6,22], and in the context of human gaze prediction see [23].) The MAE is defined in (4) for continuous saliency map S(x,y) normalized to the range [0, 1] and the binary ground-truth G(x,y) (i.e., 1 within a marked region and 0 outside), where *w* and *h* represent the width and the height of the saliency map, respectively.

(4) $$MAE=\frac{1}{w\times h}\sum_{x,y}\left|S(x,y)-G(x,y)\right|$$

The MAE provides an overall measure of the quality of a saliency map [22]. Smaller MAE values correspond to a better agreement between the salient regions and the objects in the image. Therefore, we compute the MAE between the saliency maps obtained with different bottom-up models and the binary ground-truth masks containing the traffic participants in the image.

### 3.4. Overall Proportion of Salient Areas

**Figure 4:**
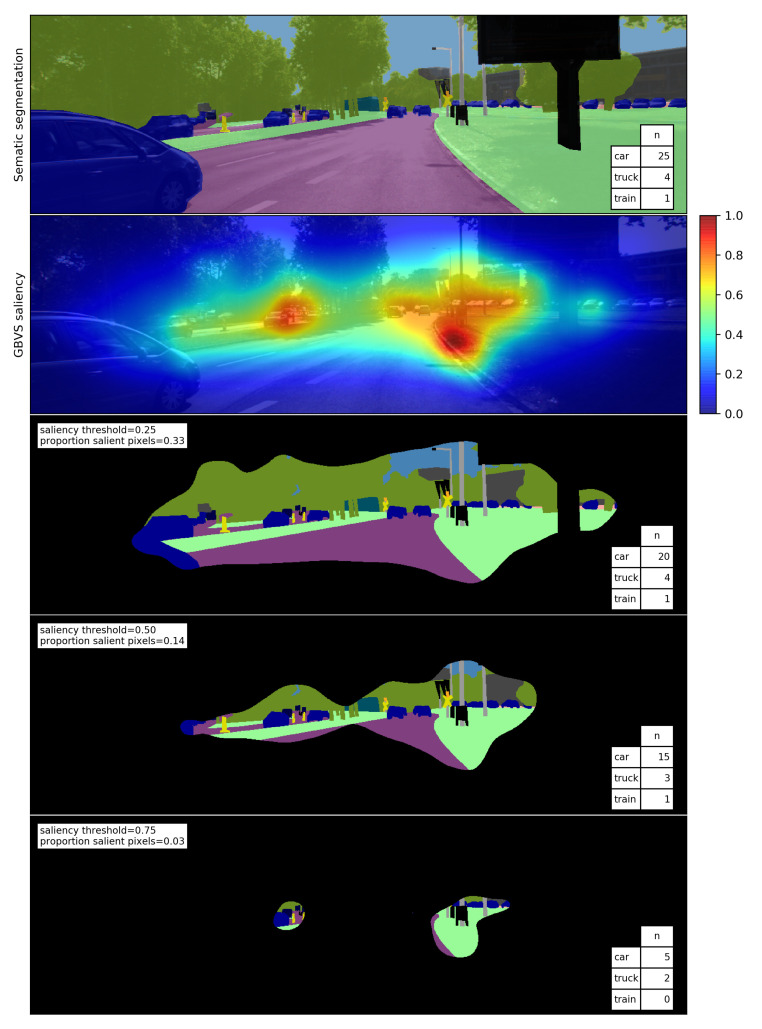
Example of the threshold analysis for a given segmented image. The top image shows the segmented image and the number *n* of object instances. Below the corresponding saliency map computed with the GBVS model is displayed. Subsequently, the proportion of salient pixels and the number of object instances in the salient area for different saliency thresholds are shown.

Saliency maps computed with different models differ in the size of the areas identified as salient. We analyze how much of the image is salient by thresholding the saliency maps. As the threshold increases, the number of pixels in the salient area decreases. We quantify this using (5) by computing the proportion $PS_{th}$ of salient pixels for each of the following saliency thresholds th: 0.25, 0.5 and 0.75, where *n* is the number of pixels in the salient area and *N* is the total number of pixels in the image. An example of how the proportion of salient pixels decreases for different saliency thresholds is shown in Figure 4.

(5) $$PS_{th}=\frac{n}{N}$$

### 3.5. Object Instance Saliency

We analyze the extent to which object instances are contained within the salient area of the image for different saliency thresholds. As the threshold increases, the number of object pixels within the salient area decreases. In (6), we compute the proportion of salient pixels within the object instance $OPS_{th}$ for each of the following saliency thresholds th: 0.25, 0.5 and 0.75, where $n_{salient\;in\;object}$ is the number of object pixels within the salient area and $N_{object}$ the total number of object instance pixels.

(6) $$OPS_{th}=\frac{n_{salient\;in\;object}}{N_{object}}$$

To account for cases in which an object instance is partially within the salient area, the object instance is regarded as salient if $OPS_{th}>0.5$. Thus, as the saliency threshold increases, fewer object instances are salient. An example of the number of salient instances for different saliency thresholds is shown in Figure 4.

## 4. Results

### 4.1. Energy and Entropy Comparison of Saliency Maps

**Figure 5:**
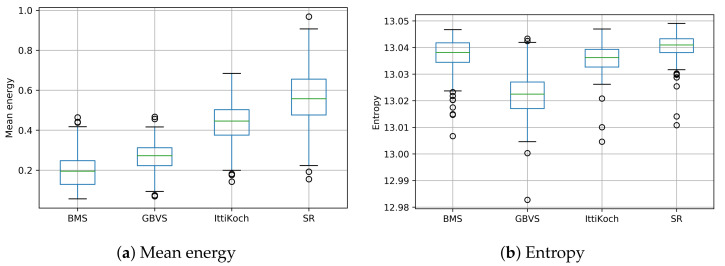
Mean energy and entropy of the saliency maps computed with different models.

The distribution of the mean energy values obtained with the selected models is shown in Figure 5a. There are significant differences in the mean energy of the saliency maps computed with different models under a repeated measures ANOVA test [24] F(3,796)=4.632, p<0.001. A post-hoc Tukey test [25] (see Table 1) revealed that all the pairwise differences are significant (p<0.001). These results indicate that the number of salient regions and their shape complexity differ across models (cf. Figure 1). Although BMS produces more simple-shaped salient areas, SR produces a larger number of complex-shaped salient areas (cf. Figure 2).

The distribution of the entropy values obtained with the selected models is shown in Figure 5b. There are significant differences in the entropy of the saliency maps computed with different models under a Friedman test [26] $\chi^{2}\left(3\right)=352.4$, p<0.001. A post-hoc Nemenyi pairwise test [27] (see Table 2) revealed that except for the pair BMS-IttiKoch, all the pairwise differences are significant (p<0.001). These results indicate that SR produces the most complex saliency maps in terms of the number of salient areas. In contrast, the entropy values indicate that the saliency produced by the GBVS model tends to be concentrated in a single zone.

### 4.2. MAE between Saliency Maps and Traffic Participants

**Figure 6:**
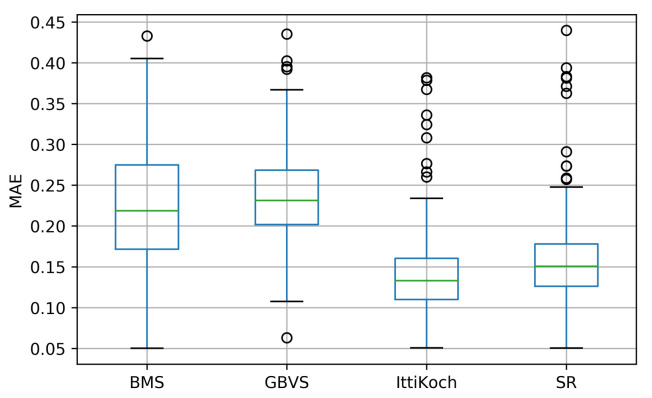
MAE between the saliency maps computed with different saliency models and the segments containing traffic participants.

The distribution of MAE values obtained with the selected models is shown in Figure 6. There are significant differences under a Friedman test: $\chi^{2}\left(3\right)=379.75$, p<0.0001. A post-hoc Nemenyi pairwise test (see Table 3) reveals that except for the pair BMS-GBVS, all the pairwise differences are significant (p<0.001). These results indicate that the best agreement (i.e., smaller MAE values) between the segments of the traffic participants and the saliency maps is obtained with the IttiKoch and SR models.

### 4.3. Comparison of the Proportion of Salient Areas

**Figure 7:**
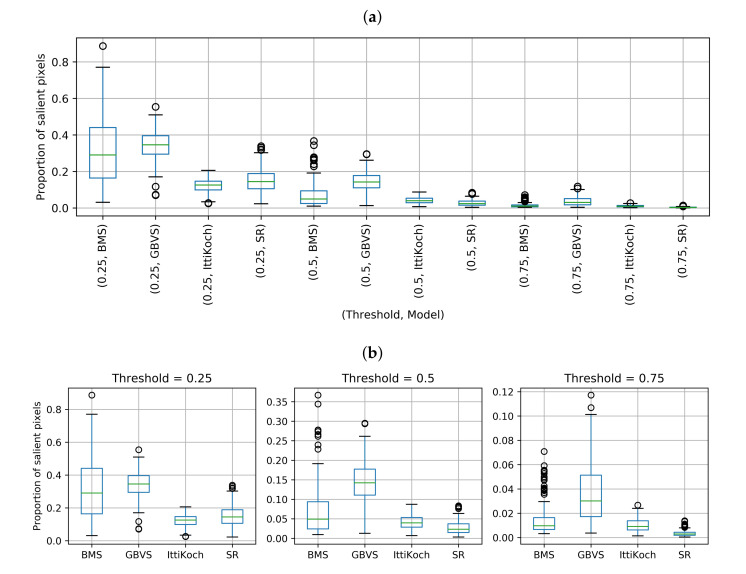
(**a**) Proportion of salient pixels over different saliency thresholds. (**b**) Detailed view over the scale of each threshold.

The proportion of salient pixels over different thresholds and saliency models is shown in Figure 7. There are significant differences under a Friedman test: $\chi^{2}\left(11\right)=1909.3$, p<0.001. The results obtained with a post-hoc Nemenyi pairwise test for the combinations of threshold and model are shown in Table 4. Note that cross-threshold pairs lack of practical meaning. Therefore, they are not interpreted in the analysis. We encounter significant differences (p<0.05), except for the following pairs: BMS-GBVS and IttiKoch-SR in the 0.25 threshold, BMS-IttiKoch in the 0.5 threshold, and BMS-IttiKoch in the 0.75 threshold. These results indicate that for the 0.5 and 0.75 thresholds the SR and GBVS models produce the smallest and largest proportion of salient pixels, respectively. Based on these results, it is expected that the number of objects within the salient areas differs across the models. Therefore, in the following section, we perform a thresholding analysis to determine the extent to which object instances are contained within the salient areas of an image.

### 4.4. Comparison of Object Instance Saliency

The percentage of salient object instances with respect to the total number of object instances in the dataset for different saliency thresholds is shown in Table 5. The table indicates that for all the objects, the largest percentage of salient instances is identified with the GBVS model for all the saliency thresholds. The results shown for the rider, bus, train, motorcycle, caravan, and trailer should be interpreted with caution due to the low number of instances with respect to the total number of images in the dataset.

### 4.5. Qualitative Analysis

Based on the quantitative results shown in the previous sections we perform a qualitative analysis of the saliency maps obtained with different models. This analysis aims to illustrate the cases in which the saliency models show the best and worst agreement with respect to the segments of traffic participants, highlighting the advantages and shortcomings of each model.

#### 4.5.1. Qualitative Analysis BMS Saliency Maps

**Figure 8:**
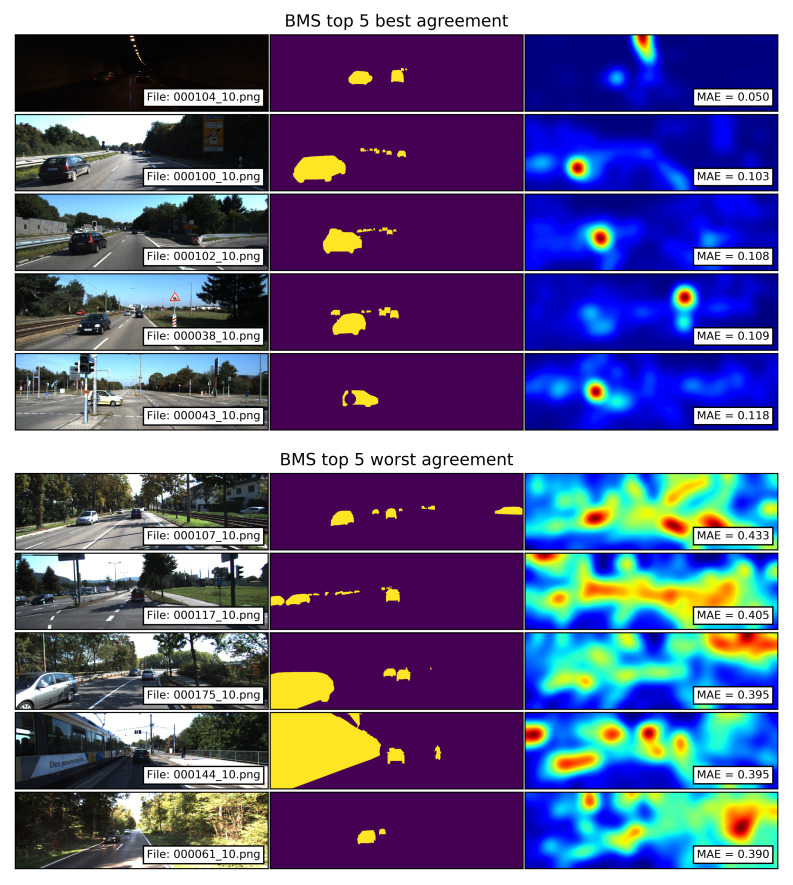
Top 5 best and worst agreements between the segments of traffic participants and the saliency maps computed with the BMS model. Smaller MAE values correspond to a better agreement.

The top 5 best and worst agreements between the segments of traffic participants and the saliency maps computed with the BMS model are shown in Figure 8. The saliency maps with the best agreements show that while smaller traffic participants in the background fall within a uniform salient area, the mid-size vehicles in the foreground generate high-saliency blobs. The simple-shaped high-saliency areas can be attributed to the closed contours of the vehicles. It is important to note that even in the pictures with small MAE values, elements such as lights (image 000104_10.png ) and traffic signs (images 000038_10.png and 000043_10.png) generate areas of higher salience compared to the ones located over the traffic participants.

The saliency maps with the worst agreements show that even though smaller traffic participants in the background fall within salient areas, elements in the background such as tree branches and the grassy field form closed contours which are identified by the model as salient. Such spurious salient areas lead to a large MAE. The image with the train (000144_10.png) shows another way in which closed contours can generate large MAE values. The closed shapes formed by the train’s windows and its white side generate high-saliency blobs, which result in a non-uniform saliency distribution over the ground-truth segment.

#### 4.5.2. Qualitative Analysis of GBVS Saliency Maps

**Figure 9:**
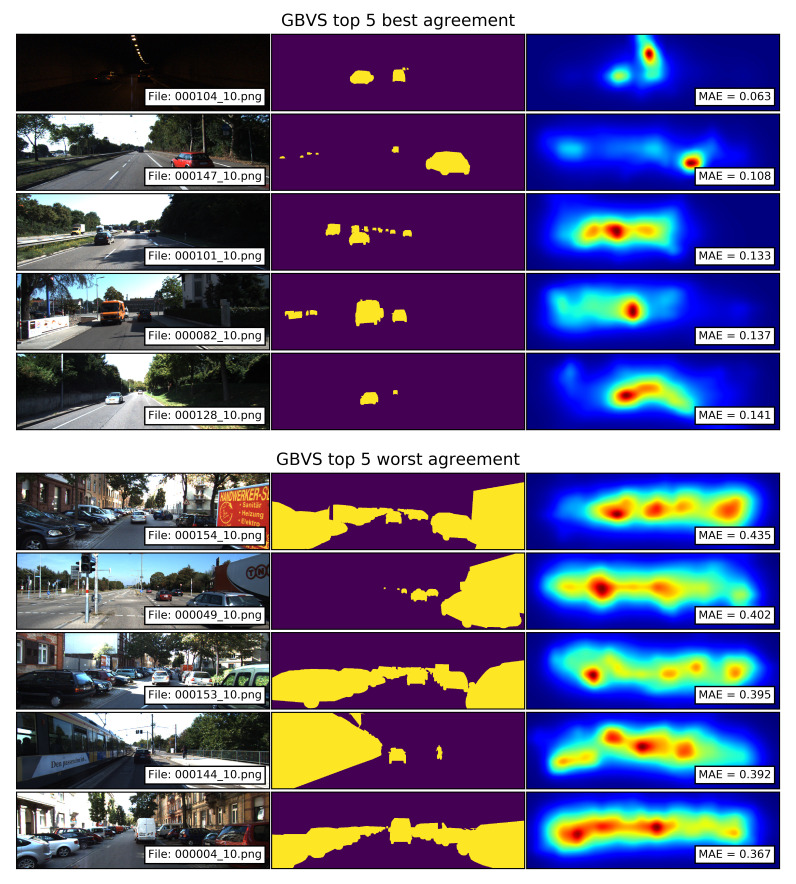
Top 5 best and worst agreements between the segments of traffic participants and the saliency maps computed with the GBVS model. Smaller MAE values correspond to a better agreement.

The top 5 best and worst agreements between the segments of traffic participants and the saliency maps computed with the GBVS model are shown in Figure 9. The saliency maps with the best agreements show that the model produces a good match for mid- and small-size traffic participants located in the central-horizontal regions of the image. This result can be attributed to the center bias of the model. The examples also illustrate how the model groups sparse edges into regions (images 000101_10.png and 000128_10.png), by which small-sized vehicles in the background fall into high-saliency blobs. The saliency maps with the worst agreements show that large segments of traffic participants located at the extreme sides of the image, such as parked vehicles or vehicles approaching from the sides, fall out of the salient areas resulting in large MAE values. This can also be attributed to the center bias of the model.

#### 4.5.3. Qualitative Analysis of IttiKoch Saliency Maps

**Figure 10:**
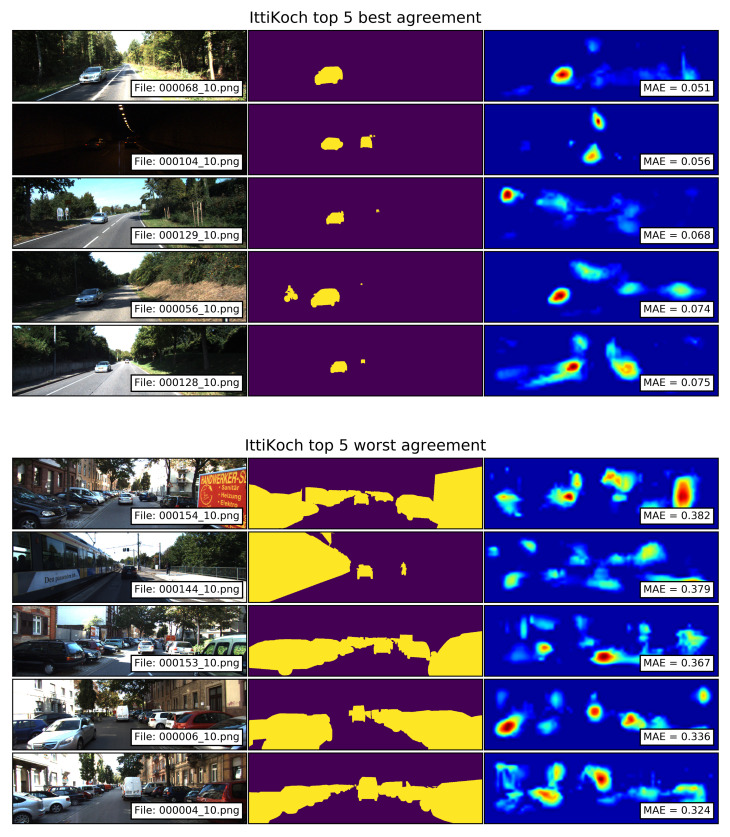
Top 5 best and worst agreements between the segments of traffic participants and the saliency maps computed with the IttiKoch model. Smaller MAE values correspond to a better agreement.

The top 5 best and worst agreements between the segments of traffic participants and the saliency maps computed with the IttiKoch model are shown in Figure 10. The saliency maps with the best agreements show that images with mid-sized traffic participants which locally stand out from their surroundings produce small MAE values. The saliency maps with the worst agreements show that as in the case of GBVS, large segments of traffic participants located at the extreme sides of the image fall out of the salient areas resulting in large MAE values. In this case, the center–surround contrast operation highlights only small portions of the vehicles, which results in separated salient areas.

#### 4.5.4. Qualitative Analysis of SR Saliency Maps

**Figure 11:**
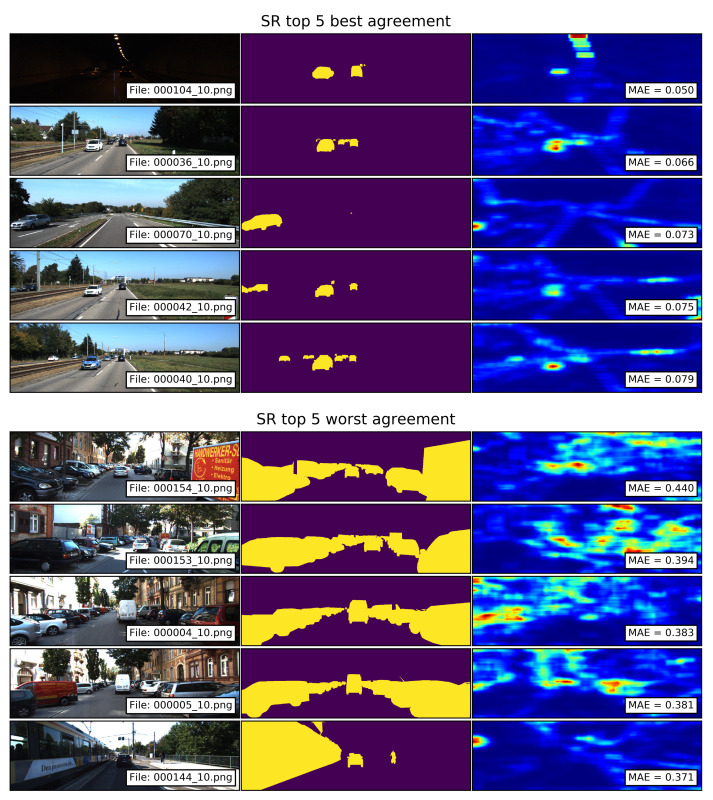
Top 5 best and worst agreements between the segments of traffic participants and the saliency maps computed with the SR model. Smaller MAE values correspond to a better agreement.

The top 5 best and worst agreements between the segments of traffic participants and the saliency maps computed with the SR model are shown in Figure 11. The saliency maps with the best agreements show that mid- and small-sized traffic participants located in the central-horizontal regions of the image fall within the salient regions. The saliency maps with the worst agreements show that as in the case of GBVS and IttiKoch, large segments of traffic participants located at the extreme sides of the image produce large MAE values. In these examples, the parked vehicles produce small salient areas of complex shapes corresponding to the boundaries of the vehicles. In addition to this, background elements such as trees and buildings produce spurious high-saliency areas.

## 5. Discussion

In this paper, we conducted a systematic evaluation of the saliency maps computed with different bottom-up models. The number of salient areas and their shape-complexity was analyzed by comparing the mean energy of the saliency maps. Additionally, whether saliency is distributed over different areas or concentrated within a single zone was analyzed by comparing the entropy of the saliency maps.

Regarding the shape complexity of the salient areas, the BMS and SR models produced simple-blob-like and complex-shaped areas, respectively. The entropy values revealed that although saliency produced by the SR model is distributed over several areas, the saliency produced by the GBVS model tends to be concentrated in a single zone.

The analysis of the MAE values revealed that the best agreements between the salient areas and the segments of the traffic participants are obtained by the IttiKoch model, followed by SR. A qualitative analysis showed that segments of traffic participants located at the extreme sides of the image, which occur when there are parked vehicles or when vehicles approach from the side, result in large MAE values with the GBVS, IttiKoch and SR models. The analysis also showed that background elements, such as tree branches and buildings, produce high-saliency regions with the BMS and SR models, which results in a reduced agreement between the salient areas and the traffic participants.

The insights about the shape and the number of salient areas were complemented by analyzing their size, quantified as the proportion of salient pixels. The comparison of this proportion over different saliency thresholds revealed that the SR and GBVS produce smaller and larger salient regions, respectively.

Given the differences between models with respect to the amount, shape and size of the salient areas, we analyzed the extent to which object instances are contained within the salient areas of an image. The analysis indicates that for the car, truck, person, and bicycle objects the largest percentage of salient instances is identified with the GBVS model across different saliency thresholds.

The analysis suggests that due to the smaller size and the shape complexity of the SR saliency maps fewer object instances fall within the salient areas of an image. In contrast, the larger size and concentration of saliency over one area of the GBVS model increases the likelihood of object instances being contained within the salient areas of an image. In this respect, it is important to recall that the salient areas of SR maps show a better agreement with the segments of traffic participants in contrast with the worse agreement obtained with GBVS. Altogether, these results constitute a trade-off between coverage and agreement for the SR and GBVS models.

It is important to note that saliency of particular object categories can be improved by means of adding a top-down prior component such as the vanishing point of the road [5] or the horizon line of the image [28]. Based on our qualitative analysis, a top-down prior could be applied to reduce the saliency assigned to trees and buildings and to increase saliency to traffic participants on the side of the image. Furthermore, the saliency of particular object categories or image areas can also be improved by combining the predictions of different bottom-up models.

The suitability of a particular model depends on the application. Although for object segmentation it is desirable that the high-saliency areas correspond to the object’s contours, for object detection large portions of the object should be located within salient areas. Furthermore, in object-detection applications for autonomous vehicles is highly important that all traffic participants fall within a predicted map because otherwise they become invisible and could lead to dangerous situations. Our evaluation method and the features used to characterize the saliency maps provide selection criteria that can be applied to different computer vision and prediction of driver behavior tasks. For example, SR saliency maps might be appropriate for a segmentation pipeline due to the large number of complex-shaped salient areas, as quantified by the entropy and energy features. On the other hand, the large and simple-shaped salient areas with center bias produced by the GBVS model can be used to prune the surveillance area of an algorithm.

To extend the insights obtained from our quantitative and qualitative results, further evaluations need to be conducted on other datasets including various light, weather and traffic conditions, which might introduce a large variability in the images registered by the frontal camera of a vehicle. However, it is important to note that such an evaluation is challenging to conduct with full control of factors to achieve a fair comparison. For example, a fair comparison between different rain intensities would require constant background and constant, or at least highly comparable, positioning of traffic participants and other elements in the scene. Furthermore, future work will be focused on the combination of bottom-up and top-down models to obtain reliable prior information for object detection and tracking algorithms such as [29]. In this way, we assume to reduce both the computational effort and the number of false detections resulting in more accurate environmental perception for autonomous driving scenarios.

## Figures and Tables

**Table 1:** Tukey multiple comparisons of means for energy. 95% family-wise confidence level.

Contrast	diff	lwr	upr	p adj
IttiKoch-BMS	0.24	0.21	0.27	<0.001
SR-BMS	0.36	0.33	0.39	<0.001
GBVS-BMS	0.07	0.04	0.10	<0.001
SR-IttiKoch	0.12	0.09	0.15	<0.001
GBVS-IttiKoch	−0.17	−0.20	−0.14	<0.001
GBVS-SR	−0.29	−0.32	−0.27	<0.001

**Table 2:** *p*-Values obtained with a Nemenyi pairwise test for entropy.

	BMS	IttiKoch	SR
IttiKoch	0.08		
SR	<0.001	<0.001	
GBVS	<0.001	<0.001	<0.001

**Table 3:** Nemenyi pairwise test for MAE.

	BMS	IttiKoch	SR
IttiKoch	<0.001		
SR	<0.001	<0.001	
GBVS	0.16	<0.001	<0.001

**Table 4:** *p*-Values obtained with a Nemenyi pairwise test for proportion of salient pixels over different saliency thresholds.

Threshold		0.25				0.5				0.75		
	Model	BMS	GBVS	IttiKoch	SR	BMS	GBVS	IttiKoch	SR	BMS	GBVS	IttiKoch
0.25	GBVS	0.78										
	IttiKoch	<0.001	<0.001									
	SR’	<0.001	<0.001	0.81								
0.5	BMS	<0.001	<0.001	<0.001	<0.001							
	GBVS	<0.001	<0.001	0.96	1.00	<0.001						
	IttiKoch	<0.001	<0.001	<0.001	<0.001	0.79	<0.001					
	SR	<0.001	<0.001	<0.001	<0.001	<0.001	<0.001	0.02				
0.75	BMS	<0.001	<0.001	<0.001	<0.001	<0.001	<0.001	<0.001	<0.001			
	GBVS	<0.001	<0.001	<0.001	<0.001	0.03	<0.001	0.91	0.72	<0.001		
	IttiKoch	<0.001	<0.001	<0.001	<0.001	<0.001	<0.001	<0.001	<0.001	1.00	<0.001	
	SR	<0.001	<0.001	<0.001	<0.001	<0.001	<0.001	<0.001	<0.001	<0.001	<0.001	<0.001

**Table 5:** Percentage of salient objects with respect to the total number of instances over different saliency thresholds. The column n shows the total number of object instances in the dataset. The saliency model with the largest percentage of salient instances within each threshold is emphasized.

		Threshold 0.25				Threshold 0.5				Threshold 0.75			
object	n	BMS	GBVS	IttiKoch	SR	BMS	GBVS	IttiKoch	SR	BMS	GBVS	IttiKoch	SR
car	810	72.8	**87.7**	12.8	39.4	20.4	**68.6**	2.1	5.8	2.6	**25.4**	0.4	0.0
truck	101	70.3	**95.0**	26.7	40.6	23.8	**87.1**	5.0	2.0	5.0	**43.6**	0.0	0.0
person	100	70.0	**86.0**	11.0	26.0	29.0	**53.0**	0.0	4.0	6.0	**18.0**	0.0	0.0
bicycle	43	53.5	**76.7**	0.0	16.3	11.6	**51.2**	0.0	0.0	0.0	**11.6**	0.0	0.0
rider	29	69.0	**86.2**	3.4	20.7	17.2	**69.0**	0.0	6.9	0.0	**20.7**	0.0	0.0
bus	19	52.6	**100.0**	10.5	42.1	26.3	**63.2**	0.0	5.3	10.5	**31.6**	5.3	0.0
train	18	72.2	**88.9**	22.2	16.7	11.1	**72.2**	5.6	5.6	0.0	**16.7**	0.0	0.0
motorcycle	8	**87.5**	**87.5**	0.0	62.5	25.0	**75.0**	0.0	12.5	0.0	**37.5**	0.0	0.0
caravan	7	**100.0**	**100.0**	28.6	57.1	28.6	**100.0**	0.0	14.3	14.3	**42.9**	0.0	0.0
trailer	5	**100.0**	60.0	20.0	40.0	0.0	**40.0**	0.0	0.0	0.0	**20.0**	0.0	0.0

## Supplementary Materials

The following are available online at https://www.mdpi.com/article/10.3390/s21206825/s1, Video S1: Saliency maps over a sequence in a traffic scene. The video illustrates the BMS, IttiKoch, GBVS and SR computed over each frame from a sequence of the KITTI object tracking dataset (sequence id: 0006).

## Abbreviations

The following abbreviations are used in this manuscript:

ADAS	Advanced Driver Assistance System
ANOVA	Analysis of Variance
BMS	Boolean Map Saliency
GBVS	Graph-Based Visual Saliency
HAD	Highly Autonomous Driving
MAE	Mean Absolute Error
SR	Spectral Residual
